# Network Analysis of How Different Physical Activity Domains Relate to Cognition in Older Adults

**DOI:** 10.1093/geroni/igaf122.3979

**Published:** 2025-12-31

**Authors:** Daniel Leme, Zhe He

**Affiliations:** Florida State University, Tallahassee, Florida, United States; Florida State University, Tallahassee, Florida, United States

## Abstract

Cognitive performance in older adults is shaped by a complex interplay of clinical and psychosocial factors. While previous studies have demonstrated bivariate associations between physical activity (PA) and cognitive decline, less is known about how distinct PA domains are simultaneously associated with mild cognitive impairment (MCI), particularly when accounting for potential confounding variables. This study examined the simultaneous associations between specific PA domains and MCI, while controlling for a broad range of clinical, psychosocial, and demographic factors, using network analysis. Data from 3,172 adults aged 60 years and older who participated in the 2011–2014 National Health and Nutrition Examination Survey (NHANES). PA was assessed using the Global Physical Activity Questionnaire (GPAQ), which classifies activity into three domains: occupational PA (OPA), including household tasks and food gathering; transportation PA (TPA), such as walking and cycling; and leisure-time PA (LTPA), encompassing sports, exercise, and recreational activities. Among participants, 10.3% reported moderate to high levels of OPA, 3.7% engaged in TPA, and 4.4% in LTPA. The final network model revealed significant negative associations between both OPA (edge weight = –0.22) and LTPA (edge weight = –0.20) with MCI. No meaningful association was found between TPA and MCI. Additionally, depressive symptoms, social isolation, and polypharmacy were positively associated with MCI (edge weights ≤ 0.22). Our findings indicate that the association between PA and MCI in older adults is domain-specific and emphasize the need for a multidimensional approach to healthy aging that integrates PA promotion with clinical and psychosocial strategies to support cognitive health.

